# The Spatial Distribution of *Dermacentor* Ticks (Ixodidae) in Germany—Evidence of a Continuing Spread of *Dermacentor reticulatus*

**DOI:** 10.3389/fvets.2020.578220

**Published:** 2020-09-25

**Authors:** Marco Drehmann, Andrea Springer, Alexander Lindau, Katrin Fachet, Sabrina Mai, Dorothea Thoma, Carina R. Schneider, Lidia Chitimia-Dobler, Michael Bröker, Gerhard Dobler, Ute Mackenstedt, Christina Strube

**Affiliations:** ^1^Department of Parasitology, Institute of Biology, University of Hohenheim, Stuttgart, Germany; ^2^Institute for Parasitology, Centre for Infection Medicine, University of Veterinary Medicine Hannover, Hanover, Germany; ^3^Bundeswehr Institute of Microbiology, Munich, Germany; ^4^Global Health Press, Marburg, Germany

**Keywords:** *Dermacentor reticulatus*, *Dermacentor marginatus*, species distribution, Germany, range expansion, citizen-science

## Abstract

In Europe, two tick species of the genus *Dermacentor* occur, *Dermacentor marginatus* and *Dermacentor reticulatus*. When the spatial distribution of both species in Germany was studied comprehensively for the first time in 1976, *D. marginatus* populations were recorded along the Rhine and Main river valleys in southwestern Germany, while *D. reticulatus* was very rare. In the last 50 years, however, a considerable range expansion of *D. reticulatus* has been noted in several European countries. To assess the current distribution of *Dermacentor* spp. in Germany, citizens were asked to send in ticks suspected to belong to the genus *Dermacentor* or that were of “unusual” appearance. From February 2019 until February 2020, 3,902 *Dermacentor* ticks were received in total. Of those, 15.48% (604/3,902) were identified as *D. marginatus* and 84.24% (3,287/3,902) as *D. reticulatus*, while 11 specimens could not be identified to species level. The majority of *D. reticulatus* specimens was collected from dogs (1,212/2,535; 47.12%), while *D. marginatus* was mostly collected from horses (184/526; 34.98%). Our results confirm that the adults of both *Dermacentor* species are active all year round. *D. reticulatus* specimens were sent in from all federal states except the Free and Hanseatic City of Hamburg, while *D. marginatus* specimens were only received from locations in southwestern Germany. Overall, data obtained from this citizen-science study show that *D. reticulatus* has significantly expanded its range, especially in northern Germany. Regarding *D. marginatus*, new locations northwest of the previous range were detected, although the distribution has remained rather stable as compared to *D. reticulatus*. The spread of *D. reticulatus*, the vector of *Babesia canis*, is of major importance for veterinarians and dog owners in terms of canine babesiosis outbreaks or endemization in hitherto *B. canis*-free areas. Thus, veterinarians and veterinary students need to be informed about the new situation to be able to give adequate advice to dog owners on the extended *D. reticulatus* range and appropriate control measures.

## Introduction

In Europe, the hard tick genus *Dermacentor* is represented by two species, *Dermacentor marginatus* (Sulzer, 1776) and *Dermacentor reticulatus* (Fabricius, 1794). The range of the ornate sheep tick, *D. marginatus*, extends from Portugal in the west throughout southern Europe and northern Africa into Central Asia. The species' southern and northern distribution limits are currently considered to be in Morocco and at the northern extension of the Rhine basin in Germany (1). Within this range, the species typically inhabits steppes, meadows, open forests, and semi-desert areas (2). The ornate dog tick, *D. reticulatus*, has a more northern distribution, occurring from northern Portugal to southern Latvia (1). It is found in a wide range of habitats, including meadows, open forests, heath-, and marshland, clearings, and suburban wasteland (3).

Immature stages of both *D. marginatus* and *D. reticulatus* are almost exclusively endophilic parasites of rodents. As adults, both sexes commonly infest larger mammals such as sheep, dogs, horses, goats, cattle (2), and occasionally humans (4). They play a role as vectors of various pathogens of considerable veterinary and medical importance. For example, both *D. marginatus* and *D. reticulatus* are competent vectors of protozoa of the order Piroplasmida, which may cause potentially fatal disease in animals. The most important causative agent of canine babesiosis in Europe, *Babesia canis*, is transmitted by *D. reticulatus* (5), while both *Dermacentor* species may transmit causative agents of equine piroplasmosis (6). Additionally, the vector function of *D. reticulatus* for tick-borne encephalitis virus (TBEV) has recently been proven (7). Although *Ixodes ricinus* (Linnaeus, 1758) is the main vector for TBEV in Europe, the virus has repeatedly been isolated from *D. reticulatus* in a TBEV-endemic area in Germany (8). Furthermore, *Dermacentor* spp. are the most relevant vectors for two causative agents of tick-borne lymphadenopathy in central Europe, *Rickettsia slovaca* transmitted by *D. marginatus* and *R. raoultii* transmitted by *D. reticulatus* (2, 3). In addition, both species are relevant in central Europe as vectors of *Francisella tularensis* (9), and *D. marginatus* may contribute to the transmission of *Coxiella burnetii*, the causative agent of zoonotic Q fever (10).

The first comprehensive study on the spatial distribution of the genus *Dermacentor* in Germany was published by Liebisch and Rahman (11). The authors reported a mosaic-like pattern of *D. marginatus* occurrence along the Rhine and Main river valleys in southwestern Germany. In contrast, an established *D. reticulatus* population was found at only one location in Germany at that time, in a forest near Tübingen. In the 1960s, *D. reticulatus* was also reported from the area of Potsdam in the former German Democratic Republic (12). In the recent past, comprehensive data on the distribution of both species in Germany have been gathered from either citizen-science approaches (13) or literature reviews (1, 14). These data, including reports on *Dermacentor* occurrence up to the year 2014, showed a considerable range expansion of *D. reticulatus*, which is in accordance with reports from other European countries, e.g., Slovakia and Poland (15, 16).

In 2019, we received indications of a further significant spread of *D. reticulatus* in Germany and thus aimed to assess the current distribution of *D. reticulatus* and *D. marginatus* in Germany by involving the general public. Citizens were asked to send in ticks belonging to the genus *Dermacentor* or of unusual appearance to allow mapping the distribution of both *Dermacentor* species in detail and identifying new areas of occurrence.

## Materials and Methods

### Citizen-Science Call

In February 2019, a single male specimen of *D. reticulatus* collected from a dog in the city of Hanover, northern Germany, was received by the Institute for Parasitology, University of Veterinary Medicine Hannover. This was an unusual finding, as hitherto the region of Hanover has not been considered within the range of this tick species. Upon request, the owner stated that the tick was found crawling on the dog after a walk and that the dog had not traveled recently. Additionally, in March 2019, a member of the institute noticed one female and three male *Dermacentor* ticks on her dog after a walk in Clausthal-Zellerfeld, located about 75 km southeast of Hanover, also hitherto not recognized as within the German *Dermacentor* range. To investigate whether these were accidental findings or if a further range expansion of *D. reticulatus* has occurred in Germany, a call to send in *Dermacentor* ticks was published in the May issue of the gazette of the Federal Chamber of Veterinarians, which is sent to every veterinarian in Germany. Furthermore, a press release was issued at the beginning of May 2019, asking citizens to send in *Dermacentor* ticks, which was shared through several print and social media.

Additionally, as of the end of February 2019, the Department of Parasitology at the University of Hohenheim near Stuttgart, southern Germany, released a call to send in *Hyalomma* ticks as well as ticks of unusual appearance. Again, respective press releases were circulated in various regional and national media, and, additionally, a website was designed for further information, where citizens were also specifically asked to send in ticks of the genus *Dermacentor*.

All media releases included pictures to help citizens distinguish between different tick genera. Along with the ticks, citizens were asked to provide information on the date and location of collection [Global Positioning System (GPS) data or postal code], the involvement of potential hosts, and details about the circumstances under which the tick was discovered. To increase motivation to participate, citizens were informed about the tick species of their specimen(s).

### Tick Identification and Geographical Classification

Ticks were identified to species level using detailed morpho-metrical keys provided by Arthur (17), Siuda (18), and Estrada-Peña et al. (19).

The accuracy of the reported locations where ticks were found was categorized based on the details provided by the senders as follows: (i) the accuracy was estimated to be high if there was a high probability that a natural habitat of the collected tick was in close proximity to the location where it was found. For example, a high accuracy was assumed for ticks collected from cattle and horses, which did not leave their pasture in the days before an infestation was detected, as well as for ticks found on vegetation, but only when the sender provided a GPS reference or precise address. (ii) A medium accuracy was assumed for unengorged ticks found on dogs or humans during or immediately after a walk, as well as for ticks from cats or wild terrestrial animals, or if the location met the criteria for a high accuracy ranking but was reported only in the form of a postal code. (iii) The reported location was considered to be of low accuracy in cases of engorged ticks found on dogs or ticks found in an unsuitable habitat (e.g. inside a house), as the origin of these ticks was often unclear. (iv) If no information on the location or the circumstances of tick detection was provided or the ticks were detected on dogs or humans travelling large distances, the accuracy was categorized as unknown.

Only locations with a high or medium accuracy were used for distribution maps. These distribution maps were compared with the results reported by Rubel et al. (1) and Naucke (13). Maps were generated in R v. 3.5.1 (20) with spatial data retrieved *via* the rworldmap package (21), *via* the eurostat package (22), and from the Global Administrative Areas Database (23).

## Results

### Tick Collection and Identification

From mid-February 2019 until the end of February 2020, 3,902 ticks of the genus *Dermacentor* were received. With a total of 3,287 (84.24%) specimens, *D. reticulatus* was sent in much more frequently than *D. marginatus* (604 specimens; 15.48%). The remaining 11 specimens (0.28%) could not be identified to species level, as essential morphological features had been destroyed. The sex ratio of *D. reticulatus* was almost 1:1 [48.65% females (1,599/3,287) vs. 51.32% males (1,687/3,287)]. In addition, one *D. reticulatus* nymph was received (0.03%). In contrast, slightly more female than male *D. marginatus* were sent in [56.79% females (343/604) vs. 43.21% males (261/604)].

### Geographic Distribution

**Figure 1:**
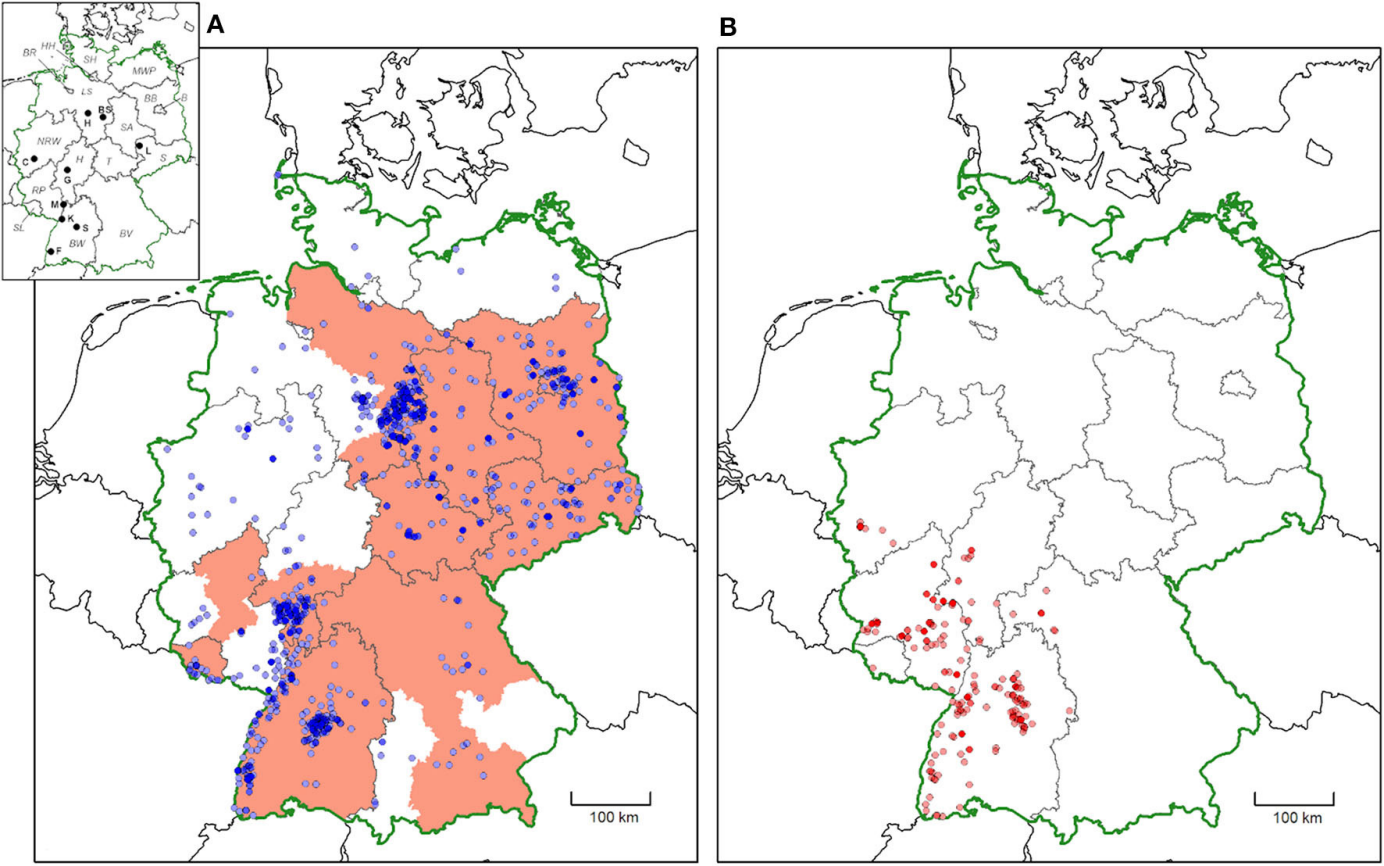
Distribution of *Dermacentor reticulatus* **(A)** and *Dermacentor marginatus* **(B)** in Germany based on ticks submitted by German citizens from February 2019 to February 2020. Only locations with medium to high accuracy are shown (*D. reticulatus*: *N* = 1,744/3,287, *D. marginatus*: *N* = 450/604). More intense colors indicate multiple findings in close proximity. Red shaded areas in **(A)** represent areas of *D. reticulatus* distribution as reported by the European Centre for Disease Prevention and Control (ECDC) in July 2019 (https://www.ecdc.europa.eu/en/publications-data/dermacentor-reticulatus-current-known-distribution-july-2019) for comparison. In the map insert, federal states are abbreviated with italic letters (B, Berlin; BR, Bremen; BW, Baden-Wuerttemberg; BV, Bavaria; BB, Brandenburg; HH, Free and Hanseatic City of Hamburg; H, Hesse; LS, Lower Saxony; MWP, Mecklenburg-Western Pomerania; NRW, North Rhine-Westphalia; RP, Rhineland-Palatinate; S, Saxony; SA, Saxony-Anhalt; SH, Schleswig-Holstein; SL, Saarland; T, Thuringia). Cities are abbreviated with bold letters (BS, Brunswick; C, Cologne; F, Freiburg; G, Gießen; H, Hanover; K, Karlsruhe; L, Leipzig; M, Mannheim; S, Stuttgart).

**Figure 2:**
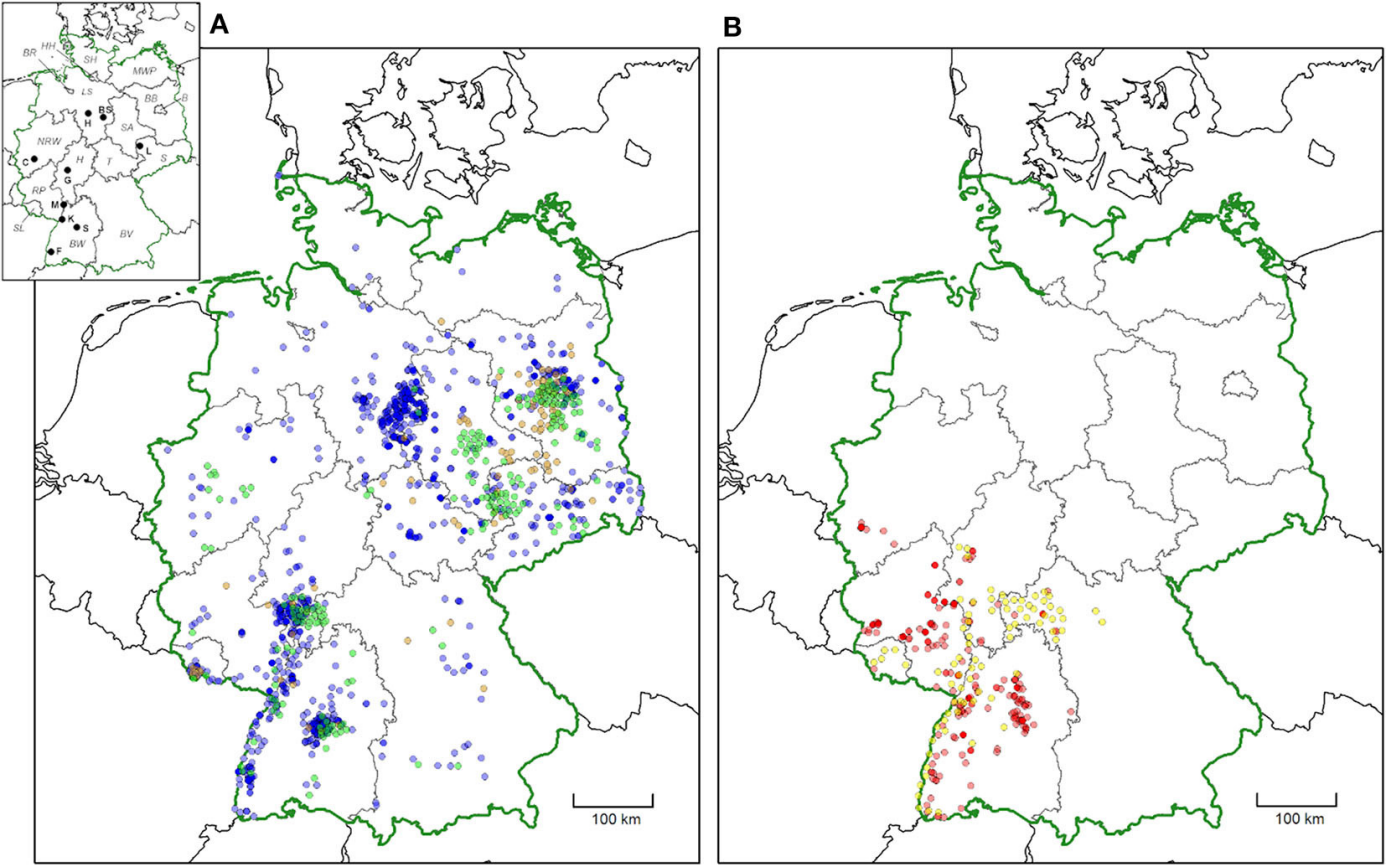
Distribution of **(A)** *Dermacentor reticulatus* (blue dots) and **(B)** *Dermacentor marginatus* (red dots) in Germany based on ticks submitted by German citizens from February 2019 to February 2020 in comparison to data from previous studies. In **(A)**, *D. reticulatus* locations as reported by Rubel et al. (1) are shown in orange and those reported by Naucke (13) in green. In **(B)** *D. marginatus* locations as reported by Rubel et al. (1) are shown in yellow. More intense colors indicate multiple findings in close proximity. In the map insert, federal states are abbreviated with italic letters (B, Berlin; BR, Bremen; BW, Baden-Wuerttemberg; BV, Bavaria; BB, Brandenburg; HH, Free and Hanseatic City of Hamburg; H, Hesse; LS, Lower Saxony; MWP, Mecklenburg-Western Pomerania; NRW, North Rhine-Westphalia; RP, Rhineland-Palatinate; S, Saxony; SA, Saxony-Anhalt; SH, Schleswig-Holstein; SL, Saarland; T, Thuringia). Cities are abbreviated with bold letters (BS, Brunswick; C, Cologne; F, Freiburg; G, Gießen; H, Hanover; K, Karlsruhe; L, Leipzig; M, Mannheim; S, Stuttgart).

For 3,877/3,902 ticks, the federal state of origin was unambiguous, whereas for 24 *D. reticulatus* and one *D. marginatus*, the federal state of origin was unclear due to travel activity of the senders. With the exception of the Free and Hanseatic City of Hamburg, *D. reticulatus* was collected in all federal states of Germany (Figure 1A). The number of *D. reticulatus* exceeded the number of *D. marginatus* received from each federal state, except for Rhineland-Palatinate, where *D. marginatus* was collected more frequently (Table 1, Figure 1B). In contrast to *D. reticulatus, D. marginatus* specimens were received from only six federal states located in southern and western Germany (Baden-Wuerttemberg, Bavaria, Hesse, North Rhine-Westphalia, Rhineland-Palatinate, and Saarland) (Table 1, Figure 1). Compared with the distribution maps provided by Rubel et al. (1) and Naucke (13), several additional sites of *D. reticulatus* occurrence are evident, especially in the north of Germany (Figure 2A). The spatial distribution of *D. marginatus* is largely comparable to the data provided by Rubel et al. (1) and Liebisch and Rahman (11). However, additional areas of occurrence were identified, for example, in the vicinity of Cologne (Figure 2B).

**Table 1:** Distribution of the 3,263 *Dermacentor reticulatus*, 603 *Dermacentor marginatus*, and 11 unidentified *Dermacentor* specimens of unambiguous origin among the federal states of Germany.

**Federal state**	***D. reticulatus***	***D. marginatus***	***Dermacentor* spp**.
Baden-Wuerttemberg	25.84% (843/3,263)	36.32% (219/603)	9.09% (1/11)
Bavaria	1.72% (56/3,263)	3.32% (20/603)	0.00% (0/11)
Berlin	1.47% (48/3,263)	0.00% (0/603)	0.00% (0/11)
Brandenburg	14.40% (470/3,263)	0.00% (0/603)	18.18% (2/11)
Free Hanseatic City of Bremen	0.03% (1/3,263)	0.00% (0/603)	0.00% (0/11)
Free and Hanseatic City of Hamburg	0.00% (0/3,263)	0.00% (0/603)	0.00% (0/11)
Hesse	7.26% (237/3,263)	7.13% (43/603)	0.00% (0/11)
Lower Saxony	18.57% (606/3,263)	0.00% (0/603)	45.45% (5/11)
Mecklenburg-Western Pomerania	0.83% (27/3,263)	0.00% (0/603)	0.00% (0/11)
North Rhine-Westphalia	1.47% (48/3,263)	2.65% (16/603)	0.00% (0/11)
Rhineland-Palatinate	2.60% (85/3,263)	50.25% (303/603)	9.09% (1/11)
Saarland	1.29% (42/3,263)	0.33% (2/603)	0.00% (0/11)
Saxony	16.24% (530/3,263)	0.00% (0/603)	0.00% (0/11)
Saxony-Anhalt	4.57% (149/3,263)	0.00% (0/603)	9.09% (1/11)
Schleswig-Holstein	0.21% (7/3,263)	0.00% (0/603)	0.00% (0/11)
Thuringia	3.49% (114/3,263)	0.00% (0/603)	9.09% (1/11)

### Temporal Course of Citizens' *Dermacentor* Collections

**Figure 3:**
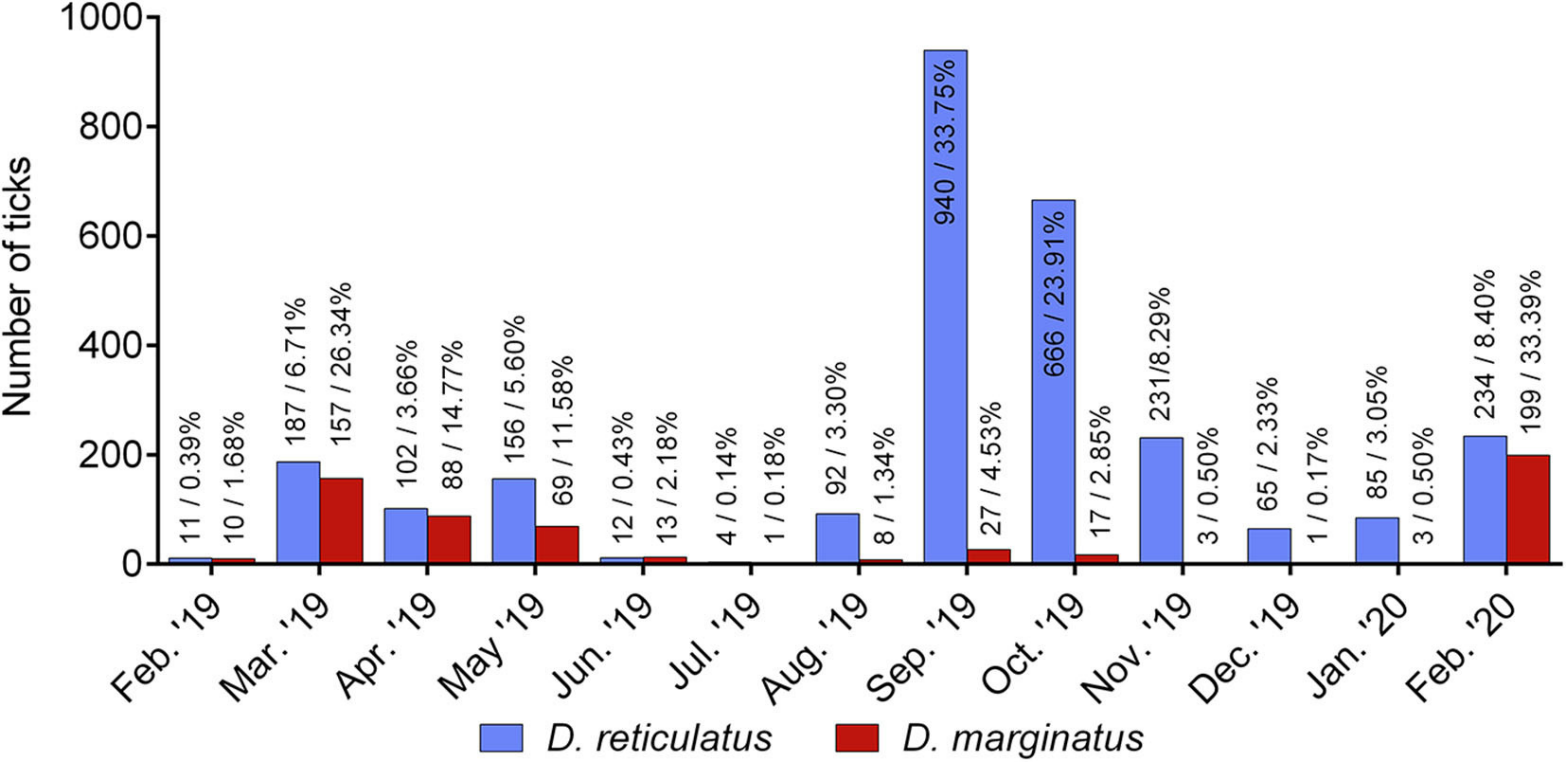
*Dermacentor reticulatus* (*N* = 2,785) and *Dermacentor marginatus* (*N* = 596) specimens by month of collection, sent in by German citizens from February 2019 to February 2020.

For 2,785/3,287 *D. reticulatus* and 596/604 *D. marginatus* specimens, information on the month of collection was provided. Both *Dermacentor* species occurred throughout the whole year. Most *D. reticulatus* specimens were found in September (940/2,785; 33.75%) and October 2019 (666/2,785; 23.91%), while smaller peaks occurred in March 2019 (187/2,785, 6.71%; following the press release by the University of Hohenheim), May 2019 (156/2,785, 5.60%; following the press release by the University of Veterinary Medicine, Hanover), and February 2020 (234/2,785, 8.40%). In comparison, *D. marginatus* numbers showed a peak in March 2019 (157/596; 26.34%) and February 2020 (199/596; 33.39%) (Figure 3).

### Host Association

Information on host association was available for 3,061/3,902 ticks (2,535/3,287 *D. reticulatus* and 526/604 *D. marginatus*). The majority of ticks were attached to or crawling on (potential) hosts, especially dogs (1,233/3,061; 40.28%) and horses (608/3,061; 19.86%). While *D. reticulatus* was collected more often from dogs (1,212/2,535; 47.81%) than from horses (423/2,535; 16.69%), *D. marginatus* was more common on horses (184/526; 34.98%) than on dogs (16/526; 3.04%). Both species were also detected on humans [*D. reticulatus*: 110/2,535 (4.34%); *D. marginatus*: 66/526 (12.55%)]. In 18 cases, citizens reported having been bitten by the ticks [nine times by *D. marginatus* (1.71%), nine times by *D. reticulatus* (0.36%)]. These ticks were often attached to the scalp. Detailed results on host association or collection location, respectively, are shown in Table 2.

**Table 2:** Host association or location of collection for the subset of *Dermacentor* ticks for which this information was available.

**Host/location**	***D. reticulatus***	***D. marginatus***	***Dermacentor* spp**.
Alpaca	0.00% (0/2,535)	0.95% (5/526)	0.00% (0/8)
Cat	0.63% (16/2,535)	0.19% (1/526)	0.00% (0/8)
Cattle	0.16% (4/2,535)	1.14% (6/526)	0.00% (0/8)
Dog	47.81% (1,212/2,535)	3.04% (16/526)	62.50% (5/8)
Donkey	0.00% (0/2,535)	29.28% (154/526)	0.00% (0/8)
Horse	16.69% (423/2,535)	34.98% (184/526)	12.50% (1/8)
Human	4.34% (110/2,535)	12.55% (66/526)	12.50% (1/8)
Moufflon	0.08% (2/2,535)	0.00% (0/526)	0.00% (0/8)
Raccoon dog	0.04% (1/2,535)	0.00% (0/526)	0.00% (0/8)
Wild boar	2.09% (53/2,535)	1.14% (6/526)	0.00% (0/8)
Car	0.24% (6/2,535)	0.57% (3/526)	0.00% (0/8)
Textiles	3.12% (79/2,535)	3.42% (18/526)	0.00% (0/8)
Garden	0.47% (12/2,535)	1.14% (6/526)	0.00% (0/8)
Indoors	5.60% (142/2,535)	7.98% (42/526)	12.50% (1/8)
Outdoors	18.74% (475/2,535)	3.61% (19/526)	0.00% (0/8)

## Discussion

The present study aimed to assess the current distribution of *Dermacentor* spp. in Germany. A continuing range expansion of *D. reticulatus* has been observed in several European countries (24–26). This range expansion has been attributed to climatic changes as well as changes in land use, travel activities of humans and pets, and an increase in available wildlife hosts, e.g., red foxes and wild boar (3). The spread of *D. reticulatus* is of considerable veterinary importance, since it is the vector of *Babesia canis*, a life-threatening protozoan blood parasite of dogs. Currently, *B. canis* transmission only occurs in restricted areas in Germany (27, 28), while autochthonous infections with other piroplasms transmitted by *Dermacentor* spp., such as *B. caballi* and *T. equi* causing equine piroplasmosis, are rare in Germany (29). Nevertheless, introduction of these pathogens with infected animals or ticks from endemic areas may lead to the emergence of new transmission foci in areas where *Dermacentor* populations are present, especially since *Babesia* spp. are transmitted transovarially in ticks (30). Furthermore, *D. reticulatus* may pose a risk for humans due to its vector role for *R. raoultii, F. tularensis*, and TBEV, among other tick-borne pathogens (2, 3).

Although *D. reticulatus* is considered to have been part of the German tick fauna for at least 100 years (31), it was limited to only few reported locations during most of the 20th century (12, 32, 33). After 1976, *D. reticulatus* apparently started spreading probably from at least two different populations, one in southwestern (11) and one in northeastern Germany (12). In the 1990s, several previously unknown *D. reticulatus* populations were described following autochthonous cases of canine babesiosis (28, 34), although comprehensive studies on the species' distribution during the last quarter of the 20th century are lacking. Since the turn of the millennium, the distribution of *D. reticulatus* in Germany has been the subject of several studies, especially with regard to its increased spread (1, 13, 32, 35, 36). As compared to field studies or literature surveys, studies involving citizens can cover a wider spatial extent and result in a larger number of records (37, 38), although the quality of the obtained data can be variable.

In the current study, only records presumably reflecting the true occurrence of the tick species, i.e., those assigned to a high or medium geographic accuracy, were taken into account for distribution maps. Our results confirm earlier studies (1, 13, 32), indicating that *D. reticulatus* is continuing its spread throughout Germany.

When compared with the most recent data provided by the European Centre for Disease Prevention and Control (39) (ECDC, cf. Figure 1A), our results show that multiple new foci of *D. reticulatus* occurrence have appeared in northern, western, and southern Germany. However, the comparability of the ECDC's map with the data generated in the present study is limited, as the resolution of the map provided by the ECDC is based on government districts and is therefore comparatively low (39).

The obtained data show notable clusters of *D. reticulatus* occurrence around the cities of Hanover and Brunswick (eastern part of the northern German federal state of Lower Saxony), Stuttgart, Mannheim, Freiburg, and Karlsruhe (western part of the southern German federal state of Baden-Wuerttemberg). In this context, however, it has to be kept in mind that citizens were asked to participate in the study *via* press releases, which were covered by various regional and national media. Regional media coverage was probably enhanced in the vicinity of the involved research institutions (located in Hanover and Stuttgart); thus, the clusters near Hanover and Stuttgart may reflect this bias in media attention. Nevertheless, *D. reticulatus* was detected for the first time in the greater Hanover area, where occurrence was also verified by flagging of questing ticks in 2019 (data not shown). In addition, the data indicate several other potentially new locations outside the hitherto known range, especially in northwestern Germany. *D. reticulatus* was even found in the northernmost part of Germany, on the island of Sylt in the North Sea (~8.34° E/54.91° N). Two independent submissions of unfed male ticks from Sylt indicate that a *D. reticulatus* population may be present on the island. However, Sylt is also a popular tourist destination, and it cannot be entirely excluded that the unfed male ticks did not originate on the island, but reached it together with traveling dog hosts.

A further cluster of *D. reticulatus* occurrence was noted in the eastern part of Germany, around the federal state of Berlin. Interestingly, the citizen-science data do not confirm the presence of *D. reticulatus* in the area between the cities of Leipzig and Berlin, where the species was previously reported (1, 32). However, this may be due to the low population density in this area, limiting the number of participants in the study. Alternatively, *D. reticulatus* may be so widespread in this region that the local population did not consider respective findings worth reporting.

The distribution of *D. marginatus* as indicated by this study is still very similar to the data presented by Liebisch and Rahman (11), also included in the distribution map by Rubel et al. (1). Nevertheless, additional locations were found in the federal state Rhineland-Palatine and in North Rhine-Westphalia, near the city of Cologne (6.96° E/50.94° N). To date, the current northern distribution limit of *D. marginatus* was believed to be near Giessen, federal state of Hesse, Germany, at coordinates 8.32° E/50.65° N (40). Thus, a slight northward spread, probably along the Rhine, did occur. Interestingly, the ecological niche model by Walter et al. (40) identified most of Rhineland-Palatinate as suitable habitat for *D. marginatus*, as well as a large area to the northeast of the distribution limit, including the entire federal state of Hesse and even the southern part of Lower Saxony. In contrast, North Rhine-Westphalia, which is located to the northwest of Giessen, was not identified as a suitable habitat by Walter et al. (40). Further studies should continue to examine whether stable populations of *D. marginatus* are permanently established in North Rhine-Westphalia.

The seasonal activity of both *Dermacentor* species in Central Europe was studied multiple times in the past (2, 3). The data presented here accord with former reports (41), as *D. reticulatus* numbers in Germany peaked in September and October and, to a lesser extent, from March to May, whereas *D. marginatus* numbers peaked in February and March. Similar patterns were observed in field studies on questing *Dermacentor* ticks in other parts of Europe [e.g. (42, 43)]. Likewise, the current study confirms winter activity of both tick species (3, 43). However, it must be kept in mind that media coverage and human behavior, among other factors, can bias data gathered by a citizen-science approach, which limits the comparability to data from field collections. Sendings in March and May 2019 were probably influenced by the preceding press releases issued by the involved research institutions.

*D. reticulatus* was found predominantly on dogs, whereas *D. marginatus* was found mostly on equids. Among domestic animals, adult *D. reticulatus* seems to prefer dogs and may even outnumber *I. ricinus* on these hosts in areas where both species occur (44). In contrast, the main hosts of adult *D. marginatus* are ungulates, especially sheep (11). The fact that no *D. marginatus* was collected from sheep in the present study may be attributed to the study design, as horse or dog owners are far more likely to notice *Dermacentor* ticks on their animals as something unusual and worth reporting than shepherds, who probably do not consider *D. marginatus* ticks as unusual. In addition, sheep are probably less often checked for tick infestation than dogs or horses, and their thick wool makes ticks hard to spot if the infestation is not severe.

In light of the zoonotic pathogens that may be transmitted by *Dermacentor* spp., it is worth noting that human tick bites were only rarely reported. Although 4.34% of *D. reticulatus* and 12.55% of *D. marginatus* for which information on host association was provided were found crawling on humans, the proportions of ticks that had actually bitten humans were only 0.36 and 1.71%, respectively. In Spain, where both *D. reticulatus* and *D. marginatus* occur, these species accounted for 2.22 and 12.52% of 4,049 ticks found on humans, respectively (4). These numbers are comparable to our data; however, no information on the proportion of ticks that had actually bitten humans appears in the Spanish study. In the areas of Liguria and Tuscany, Italy, *D. marginatus* was identified as the second most important anthropophagic tick, after *I. ricinus*, accounting for 9.1% of 565 human tick bites (45). In contrast, among 2,547 ticks removed from humans in Germany between 2013 and 2017, only 0.16% were identified as *Dermacentor* spp. (46). Overall, the available data indicate that *D. marginatus* is more likely to attach to or even bite humans than *D. reticulatus*.

## Conclusions

The present study shows that *D. reticulatus* is continuing to spread in Germany, especially in the northwestern part of the country. Overall, this tick was found in all federal states except the Free and Hanseatic City of Hamburg. In contrast, the distribution of *D. marginatus* is still restricted to southwestern Germany; however, newly identified locations in North Rhine-Westphalia show that this species has also undergone a geographical spread. A range expansion of both species is particularly worrying in light of their role as vectors. Both species may transmit human pathogens; however, they rarely seem to bite humans in Germany. Thus, the implications for public health may be considered of minor importance. By contrast, the spread of *D. reticulatus* is of major importance for veterinarians and dog owners in terms of canine babesiosis outbreaks or endemization in hitherto *B. canis*-free areas. Thus, veterinarians and veterinary students need to be informed about this situation, with updates during continuing education. Similarly, dog owners need to be advised on the expanding *D. reticulatus* range and the need for careful tick control measures by veterinarians and, where appropriate, by veterinary associations such as the German chapter of the European Scientific Counsel Companion Animal Parasites (ESCCAP).

## Data Availability Statement

The original contributions presented in the study are included in the article/supplementary material, further inquiries can be directed to the corresponding author/s.

## Author Contributions

CS, GD, UM, MD, AS, and AL designed the study. UM and CS coordinated the study and communicated with media representatives. MD designed the website. LC-D, MB, and GD contributed collected ticks. AS, MD, AL, KF, and LC-D identified tick species. AS, MD, AL, KF, SM, DT, and CRS participated in communication with the public and individual citizens as well as data handling. MD and AS drafted the manuscript. All authors participated in data interpretation, reviewed the manuscript draft, read, and approved the final manuscript.

## Conflict of Interest

The authors declare that the research was conducted in the absence of any commercial or financial relationships that could be construed as a potential conflict of interest.
